# Supplementary material for: Flatfoot and associated factors among Ethiopian school children aged 11 to 15 years: A school-based study
Source: PLoS One. 2020 Aug 25;15(8):e0238001. doi: 10.1371/journal.pone.0238001 (PMC7447044; doi:10.1371/journal.pone.0238001)
Supplement: S1 File — (DOCX) [file pone.0238001.s001.docx]

**Questionnaire ID no:**

| **SURVEY INFORMATION** |
| --- |

| **Location and Date, Consent, ID** | | **Response** | **Code** |
| --- | --- | --- | --- |
| 1. | School Name |  | **A1** |
| 2. | Type of school | Government 1 Private 2 | **A2** |
| 3. | Interviewer ID |  | **A3** |
| 4. | Date of Data collection | DD/MM/YYYY | **A4** |
| 5. | Assent &Consent has been read and obtained | Yes 1  No 2 **If No, End** | **A5** |
| 6. | Time of interview (24 hour clock) | **Hrs. Min** | **A6** |
| 7. | Reg no and Grade of Student |  | **A7** |
| 8. | Section of Class |  |  |

| **DEMOGRAPHIC INFORMATION** |
| --- |

| **Questions** | | | **Response** |  |
| --- | --- | --- | --- | --- |
| 9. | Gender (record as observed) | | Male 1Female2 | **B1** |
| 10. | Age of the student | | __ ___ ___in years | **B2** |
| 11. | Types of foot wear most of the time? | | Closed- toe shoes 1 sandals 2 | **B3** |
| 12. | Do you wear shoe when you were below 6 years or during your childhood? | | Yes 1 no 2 | **B4** |
| 13 | Which grade are you?[circle appropriate] | | Grade 5 6 7 8 | **B6** |
| **PHYSICAL MEASUREMENTS** | | | | |
| 14. | | Height in (cm) |  | C1 |
| 15. | | Weight in( kg) |  | **C2** |

| Lower extremity and foot deformities | | | | | | Identify by observation | | | | | |
| --- | --- | --- | --- | --- | --- | --- | --- | --- | --- | --- | --- |
| 16 | | Any foot deformity | | | | If there(yes 1) ,what type of deformity: | | | **D1** | | |
|  |  |  |  |  |  |  | Rt | Lt |  |  |  |
|  |  |  |  |  |  | Hallux valgus, |  |  |  |  |  |
|  |  |  |  |  |  | Hammer toes, |  |  |  |  |  |
|  |  |  |  |  |  | Heel valgus, |  |  |  |  |  |
|  |  |  |  |  |  | Knee valgus |  |  |  |  |  |
|  |  |  |  |  |  | Knee varus |  |  |  |  |  |
|  | |  | | | | Normal |  |  |  | | |
| **History of foot pain** | | | | | | | | | | | |
| 17. | Do you have foot pain now?  Have suffered foot pain in the? | | | | Present pain Yes 1 No 2  Past six months Yes 1 No 2 | | | | E1 | | |
| 18. | If yes, rate your pain? (max in six months) | | | | **0—---1—---2—---3—---4-----5** | | | | E2 | | |
| 19. | If yes, what’s the frequency of pain? | | | | 1. Rarely 2. Occasionally 3. Frequently | | | |  | | |
| 20. | If yes, what are the location of the pain? *(circle appropriate, multiple answer possible)* | | | | 1. General foot pain 8. Knee pain 2. Toe pain 9. Hip pain 3. Ball of the foot pain 10. Low back 4. Forefoot pain 5. Arch pain 6. Heel pain 7. Hind foot pain 8. Leg pain | | | | E3 | | |
| 21. | If yes, when, doing what provokes pain? *(circle appropriate, multiple answer possible)* | | | | 1. During playing 2. During walking 3. Night pain | | | | E4 | | |
| **Footprint arch index Ratio (flatfoot/normal) STAHELI PLANTAR ARCH INDEX** | | | | | | | | | | | |
| 22. | Foot print value (print id: )   \| Left \|  \| \| --- \| --- \| \| Right \|  \| | | | **PI = (A / B)Whereas, A** = support width of central region to the foot **B =** support width of heel region | | | | | | | **F1** |
| 23 | 1. Foot morphology | | Flat foot: Yes 1 No 2 | | | | | | | **F2** | |
|  | 1. Type of flat foot | | Flexible 1 Rigid 2 | | | | | | | **F3** | |
|  | 1. Site of the flat foot | | Right side 1 Left side 2 Both 3 | | | | | | | **F4** | |
| 24. | Physical activity level in atypical week | | *On the last weekend*, how much times did you do moderate and vigorous physical activities in minute/week? Like sports football, (playing hard, running, jumping, throwing_______min | | | | | | | **F5** | |
