# Supplementary material for: Flatfoot and associated factors among Ethiopian school children aged 11 to 15 years: A school-based study
Source: PLoS One. 2020 Aug 25;15(8):e0238001. doi: 10.1371/journal.pone.0238001 (PMC7447044; doi:10.1371/journal.pone.0238001)
Supplement: S2 File — (DOCX) [file pone.0238001.s002.docx]

# Title: Flatfoot and associated factors among Ethiopian school children aged 11 to 15 years: a school-based study

# STROBE Statement—checklist of items that should be included in reports of *cross-sectional studies*

|  | | Item no | | Recommendation |
| --- | --- | --- | --- | --- |
| **Title and abstract** | | 1 | | 1. Study design is indicated in the abstract, methods section as an school-based cross-sectional study design. |
|  |  |  |  | (b) A balanced summary of what was done and what was found is provided in the methods and result section of the abstract. |
| Introduction | | | | |
| Background/rationale | | 2 | | The scientific background and rationale for conducting this study is reported under background section in this manuscript. |
| Objectives | | 3 | | Specific objectives and the need for this study is stated in the last paragraph under background section. |
| Methods | | | | |
| Study design | | 4 | | This study used a school-based cross-sectional design and the same is mentioned 1^st^ paragraph of method section. |
| Setting | | 5 | | A detailed description of the study area, sample catchment area, location, population attendance in the institution, and participant, described in the first paragraph of method section under study design sub-section. |
| Participants | | 6 | | Eligibility criteria, the sources and methods of selection of visually impaired adult participants are clearly stated in the last paragraph of methods section. **(School children aged 11- 15 years)** |
| Variables | | 7 | | Both outcome (Staheli index) and predictor variables are operationally defined under methods section, data collection tools and procedures, in the 3^rd^ paragraph. |
| Data sources/ measurement | | 8 | | Source of data and data analysis methods are discussed in the 6^th^ paragraphs of methods section, under sub-section data processing and analysis sub-section. |
| Bias | | 9 | | Efforts to address potential sources of bias were described in several part of methods section. |
| Study size | | 10 | | Flow of study is described in figure 1. Additionally, sample size determination, assumptions, and sampling technique are mentioned under sample size and sampling technique sub-section under methods. |
| Quantitative variables | | 11 | | All quantitative variables treated as qualitative after categorizing them in one of most commonly used categories and category used are mentioned under data processing and analysis sub-section. |
| Statistical methods | | 12 | | (*a*) Statistical methods used in this study are described under data analysis sub-section in the last paragraph of method session. |
|  |  |  |  | (*b*) Both sub group analysis and interaction terms were used. |
|  |  |  |  | (*c*) There were no missing data in this study |
|  |  |  |  | *(d)* Not applicable |
|  |  |  |  | (*e*) Not applicable |
| Results | | | | |
| Participants | 13 | | (a) Number of school children aged 11 – 15 years, response rate and reasons for non-response are presented in the first paragraph of results session and detail socio-demographic characteristics in the table 1 and text under results. | |
|  |  |  | (b) About 97.4% children obtained parental consent and the most common reason for non-response was absence of parental consent letter, not willing to do foot ink print. | |
|  |  |  | (c) This was cross-sectional study so; there is no flow as that of longitudinal study. | |
| Descriptive data | 14 | | (a) Characteristics of study participants (eg demographic, anthropometrics, foot wear related variables, pain, social) and information on exposures and potential confounders is presented in tables 1. | |
|  |  |  | (b) There were no missing data in this study | |
| Outcome data | 15 | | Outcome variable (Flatfoot **(Staheli arch index > 1.15)**) described and summarized in table 1and 3. | |
| Main results | 16 | | (*a*) Unadjusted estimates and confounder-adjusted estimates and their precision (eg, 95% confidence interval) are presented in table 3. Discussed in 1^st^paragraph under regression analysis of result section. | |
|  |  |  | 1. Category boundaries of continuous variables were categorized and reported in all tables. | |
|  |  |  | (*c*) Regression model was used and expressed in odds ratio. | |
| Other analyses | 17 | | When a clear sub group existed, analyses were performed and interaction terms were used to examine potential association between BMI*foot pain and reported in table 3. | |
| Discussion | | | | |
| Key results | 18 | | Key results to study objectives are discussed under discussion session with references. | |
| Limitations | 19 | | Limitations and possible strengths related to this study are discussed in the final paragraph of discussion session on the way of viewing direction for researchers. | |
| Interpretation | 20 | | A cautious overall interpretation of results considering objectives, results from similar studies, and other relevant evidence is discussed under limitation of discussion session. | |
| Generalisability | 21 | | Generalizability (external validity) of the study results are mentioned in the paragraph of discussion section and in conclusion section. | |
| Other information | | | | |
| Funding | 22 | | Information regarding the source of funding **(University of Gondar, Grant no: SOM 1251/2019)** and the role of the funders for the present study is presented under acknowledgment section. | |
