# Supplementary material for: Flatfoot and associated factors among Ethiopian school children aged 11 to 15 years: A school-based study
Source: PLoS One. 2020 Aug 25;15(8):e0238001. doi: 10.1371/journal.pone.0238001 (PMC7447044; doi:10.1371/journal.pone.0238001)
Supplement: S3 File — (DOCX) [file pone.0238001.s003.docx]

**Additional table- 1 Range and mean of Staheli plantar arch index (SPAI) foot print value and lateralization of flatfoot**

| **SPAI value** | | | **Site of the flat foot** | |
| --- | --- | --- | --- | --- |
| **Right foot** | | **Left foot** |  |  |
| Range | 0.18-1.9 | 0.18-1.9 | Right side | 58 (7%) |
| Mean | 0.74 | 0.68 | Left side | 27 (3.3%) |
|  |  |  | Bilateral | 60 (7.3%) |
|  |  |  | Flat foot (summary index) | 145(17.6%) |
|  |  |  | No flat foot | 680 (83.7%) |

| **Age** | **N** | **SPAI mean** ± (range) | |
| --- | --- | --- | --- |
|  |  | **Right foot** | **Left foot** |
| 11 | 113 | 0.88±0.30 (0.40-1.71) | 0.81±0.30 (0.40-1.80) |
| 12 | 172 | 0.77±0.34 (0.20-1.80) | 0.71±0.36 (0.23-1.90) |
| 13 | 169 | 0.77±0.38 (0.22-1.75) | 0.68±0.33 (0.20-1.75) |
| 14 | 198 | 0.70±0.35 (0.18-1.90) | 0.67±0.32 (0.18-1.80) |
| 15 | 171 | 0.65±0.34 (0.21-1.80) | 0.61±0.32 (0.24-1.80) |
| **Overall** | **823** | **0.74±0.35** (0.18-1.90) | **0.69±0.33** (0.18-1.90) |
| **Test of linearity- between groups** | **823** | **F 7.06, P 0.000** | **F29.7, p 0.000** |

**Table 2 Correlation coefficient (r) values of age, height, weight, BMI, and Staheli plantar arch index (SPAI) of gender school children, Gondar town, Ethiopia (n= 823)**

| **Correlations** | | | | | | | |
| --- | --- | --- | --- | --- | --- | --- | --- |
|  | | **Age** | **Height** | **Weight** | **BMI** | **SPAI Right** | **SPAI Left** |
| **Age** | *r* | 1 | .555**^**^** | .579^**^ | .356^**^ | -.187^**^ | -.174^**^ |
|  | Significance |  | .000 | .000 | .000 | .000 | .000 |
|  | N | 823 | 823 | 823 | 823 | 823 | 823 |
| **Height** | *r* | .555^**^ | 1 | .667^**^ | .119^**^ | -.048 | -.102**^**^** |
|  | Significance | .000 |  | .000 | .001 | .170 | .003 |
|  | N | 823 | 823 | 823 | 823 | 823 | 823 |
| **Weight** | *r* | .579^**^ | .667^**^ | 1 | .813^**^ | .089^*^ | .029 |
|  | Significance | .000 | .000 |  | .000 | .010 | .407 |
|  | N | 823 | 823 | 823 | 823 | 823 | 823 |
| **BMI** | *r* | .356^**^ | .119^**^ | .813^**^ | 1 | .154**^**^** | .113^**^ |
|  | Significance | .000 | .001 | .000 |  | .000 | .001 |
|  | N | 823 | 823 | 823 | 823 | 823 | 823 |
| **SPAI Right** | *r* | -.187^**^ | -.048 | .089^*^ | .154^**^ | 1 | .783**^**^** |
|  | Significance | .000 | .170 | .010 | .000 |  | .000 |
|  | N | 823 | 823 | 823 | 823 | 823 | 823 |
| **SPAI Left** | *r* | -.174^**^ | -.102^**^ | .029 | .113**^**^** | .783^**^ | 1 |
|  | Significance | .000 | .003 | .407 | .001 | .000 |  |
|  | N | 823 | 823 | 823 | 823 | 823 | 823 |
| r*- Correlation coefficient, BMI-Body Mass Index, **. Correlation is significant at the 0.01 level (2-tailed), *. Correlation is significant at the 0.05 level (2-tailed).* | | | | | | | |
