# Supplementary material for: Flatfoot and associated factors among Ethiopian school children aged 11 to 15 years: A school-based study
Source: PLoS One. 2020 Aug 25;15(8):e0238001. doi: 10.1371/journal.pone.0238001 (PMC7447044; doi:10.1371/journal.pone.0238001)
Supplement: Attachment — Submitted filename: Response to reviewers.docx [file pone.0238001.s004.docx]

**Date: July 2020**

# POINT-BY-POINT RESPONSE LETTER

PLOS ONE

# Manuscript ID PONE-D-20-14474 Title: Flatfoot and associated factors among Ethiopian school children aged 11 to 15 years: a school-based study. We express our sincere appreciation to the reviewers and editor for their time, comments, suggestions, and interest in this study. The reviewers and editor had pointed out constructive comments, and legitimate questions which improved the paper.

Please find our responses below:

***Note: Correction and edition made in the track change version:***

|  | **(Reviewer-1) Review questions/comments** | **Response** |
| --- | --- | --- |
| **1** | This manuscript was well written and the data analysis was more than adequate. I fully agree that more attention needs to be given to the pathology of pediatric flatfeet and that they should not be considered "normal."  There were a few spacing errors and missing "." but that would be caught later in the editing process. I feel this paper is ready to proceed to the next step. | **Thank you, for your comments and time consideration.**  **We have done copy editing again to correct the spacing errors in the manuscript.** |
|  | **(Reviewer-2) Review questions/comments** | **Response** |
| **1.** | The introduction is clear and nicely written. Methods are overall sound and the statistical analysis is appropriate. Results are well presented as well. In discussion there are multiple typos which need to be addressed. Apart from this, concepts are clear. | **Thank you, for your comments and time consideration.** |
| **2.** | My only remark would be to mention further methods to define flatfoot, which is still a matter of debate, such as the Denis classification and the Cavanagh one which are mentioned in the paper  "The role of arthroereisis of the subtalar joint for flatfoot in children and adults." EFORT Open reviews 2017, highlighting the differences and the advantages of one method as compared to one other. | **Yes, thank you for pointing out the gap.**  **We have mentioned about the other footprint methods under the 3^rd^ para of discussion section now. Page 19-20, Line 392-397**  **We have amended the suggestion in 3^rd^ para under discussion section highlighting the arthroeresis procedure and advantage of using weight-bearing radiographs over the footprint methods. Page 19, Line 389-392**  **Thank you for the comments and helping with the reference paper as well.** |

With regards

Balamurugan Janakiraman
